# Sectoral Ciliary Body Agenesis Complicated with Cataract Formation Diagnosed by Ultrasound Biomicroscopy

**DOI:** 10.4274/tjo.78535

**Published:** 2018-12-27

**Authors:** Özgün Melike Gedar Totuk, İlhami Salcan, Melih Atalay, Ümit Aykan

**Affiliations:** 1 Bahçeşehir University Faculty of Medicine, Department of Ophthalmology, İstanbul, Turkey; 2 Özel Avrupa Hospital Arnavutköy, Ophthalmology Clinic, İstanbul, Turkey; 3 Bahçeşehir University Faculty of Medicine, İstanbul, Turkey; 4 Dünyagöz Hospital, Ophthalmology Clinic, İstanbul, Turkey

**Keywords:** Ciliary body agenesis, coloboma, ultrasound biomicroscopy

## Abstract

We aimed to present a novel case of sectoral ciliary body agenesis and complicated cataract as an embryogenic defect of eye development diagnosed by ultrasound biomicroscopy. A 20-year-old male patient presented with a complaint of visual impairment in his left eye since childhood. Slit-lamp examination of the left eye revealed pigment precipitation and focal lens opacities extending from the temporal quadrant through the posterior lens capsule, blocking the central optical axis. On ultrasound biomicroscopy examination, there was a hyperechoic reflection belonging to the rudimentary ciliary body structures between 2-5 o’clock in the temporal quadrant. The zonules could not be visualized in the same location. At all other quadrants of the anterior chamber angle, the ciliary body and zonules were normal. This is a very rare case of sectoral ciliary body agenesis complicated by cataract. Ultrasound biomicroscopy may be useful for detecting rare congenital anomalies of the anterior segment, anterior chamber angle, and ciliary body.

## Introduction

The embryological development of the human eye involves a series of events, beginning with fertilization of the ovum and continuing through the early postnatal period, in three embryonic layers: neural ectoderm, neural crest, and surface ectoderm, with minor contribution from the mesoderm.^1^ The lens arises from the surface ectoderm and formation starts with the contact of optic vesicle at about 3 weeks of gestation.^2^ The cranial neural crest is the origin of the ciliary body, including pigmented and nonpigmented cells and the ciliary smooth muscle, and it starts to form in the third month as a fold posterior to the progressing edge of the optic cup.^3,4^ Developmental defects in embryogenesis cause ocular malformations in a spectrum from congenital cataract to anophthalmia.^5^

In this report, we aimed to present a novel case of sectoral ciliary body agenesis and complicated cataract as an embryogenic defect of eye development diagnosed by ultrasound biomicroscopy (UBM).

## Case Report

A 20-year-old male patient presented to our clinic with a complaint of visual impairment in his left eye since his childhood. The patient had no ocular or systemic disease, history of trauma, ophthalmic surgery, or chronic medication. In detailed ophthalmic examination, best corrected visual acuity (BCVA) in the right eye was 10/10 with Snellen chart and anterior and posterior segment evaluation was normal. BCVA in the left eye was limited to hand motions. His eyes were orthophoric in primary position, and there was no restriction of eye movements. Pupillary light reactions were normal. Intraocular pressure measured by applanation tonometry was 13 mmHg in the right eye and 12 mmHg in the left eye.

**Figure 1:**
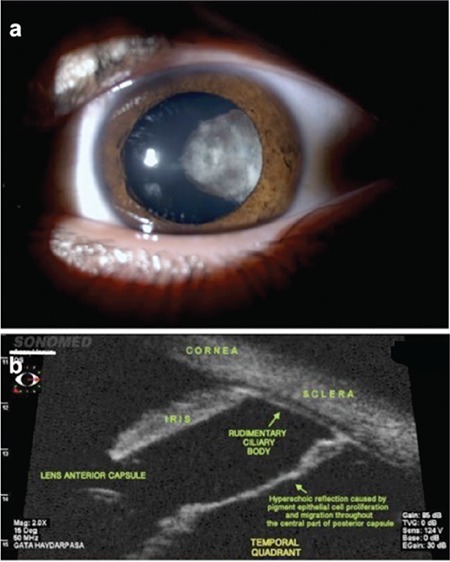
a) Anterior segment photography of the left eye. b) Radial angle image of the temporal quadrant of the left eye on ultrasound biomicroscopy

Slit-lamp examination of the left eye revealed pigment precipitation and focal lens opacities extending from the temporal quadrant through the posterior lens capsule and blocking the central optical axis (Figure 1a).

On UBM examination, there was a hyperechoic reflection belonging to the rudimentary ciliary body structures between 2-5 o’clock in the temporal quadrant. The zonules could not be visualized in the same location (Figure 1b). In all other quadrants of the anterior chamber angle, the ciliary body and zonules were normal. Media opacities prevented a full fundoscopic examination.

## Discussion

Ocular development starts in the third week of gestation. Any error in this process can cause congenital ocular malformations.^6^ In terms of ocular developmental chronology, there’s no direct connection between formation of the ciliary body and lens other than their location. Embryologically, the ciliary body consists of two different tissue layers. The pigmented and nonpigmented ciliary epithelium derives from the neuroectoderm, while the ciliary muscles and the stroma derive from the neural crest cells. The lens is derived from the surface ectoderm. Similarly, lens formation begins in the third week of gestation as the “lens placode” and initial development concludes in the seventh week. Ciliary body formation starts in the third month of gestation and ends in the fifth month^1^. Apart from their physical proximity, another possible connection is the arrival of growing ciliary processes at the equator and formation of lens zonules in the fourth month. Therefore, it is thought that in our case developmental defect occurred primarily in ciliary body and the lens was affected indirectly. 

UBM performed in our patient did not reveal any significant echogenicity belonging to zonules at the defective site. The hypoplastic, rudimentary, underdeveloped ciliary body and lack of connection between the ciliary processes and lens equator, which must have been there since the fourth month of gestation, explains the absence of the zonules.

Other than aqueous humor secretion and accommodation, the ciliary body has another vital function: nutrition of the lens.^7^ Interestingly, in our case, only the defect site had focal lens opacities. This may be related to metabolic defects of the lens. However, in that situation, these opacities should be present throughout the entire lens. We also believe that the central pigment precipitation extending through the posterior lens capsule at the defect site might be the more likely reason for formation of these opacities.

Chronologically, the pigmented and nonpigmented ciliary epithelial tissue develop first, followed by the ciliary processes and zonules, and finally the pars plana, ciliary body stroma, and ciliary muscles.^4^ This sequence supports the absence of any affected tissue other than the defective ciliary pigment layer in our case.

It is clear that there is a difference between prenatal and postnatal development of nasal and temporal ciliary bodies.^8,9^ This difference supports the temporal ciliary body defect site seen in our case. Why the temporal quadrant is affected rather than the nasal quadrant is another pressing question that has yet to be answered.

Because this case was unilateral and the defective ciliary body was not in the closure site of the embryonic fissure, there was a slight possibility of atypical coloboma. However, the presence of normal ocular structures other than the ciliary body reduced this possibility.

UBM is useful in a variety of clinical applications in the diagnosis of eye diseases, providing detailed cross-sectional anterior chamber anatomy with high resolution and reproducible images. In contrast to anterior segment optical coherence tomography and Scheimpflug imaging, which do not penetrate past the iris pigment epithelium to a large degree, UBM can show the exact configuration and position of the iris, ciliary body, and ciliary processes even in the presence of opaque media.^10^ In our case, we diagnosed ciliary body sectoral agenesis by means of UBM.

To the best of our knowledge, there is no report similar to our case in the English literature. In the near future it will be possible to evaluate cases like these more reliably due to enhanced imaging techniques, developing technology, and current embryologic research. Genetic analysis to investigate mutational defects, which might be associated with sectoral ciliary body agenesis, could offer valuable information.

In conclusion, this is the first report of sectoral ciliary body agenesis complicated by cataract in the English literature. UBM may be useful for detecting rare congenital anomalies of the anterior segment, anterior chamber angle, and ciliary body.
